# 

**Published:** 1902-02-08

**Authors:** 


### NOTICE TO CORRESPONDENTS.

All MSS., Letters, Books for Review, and other matters intended for the Editor should be addressed The Editor, "The Hospital," The Hospital Building, 28 & 29 Southampton Street, Strand, W.C.

The Editor cannot undertake to return rejected MS., even when accompanied by stamped directed envelope.

**Notice to Advertisers and Subscribers.**

All Advertisements, Orders for Copies of The Hospital, and Business Communications should be addressed to **THE MANAGER (Not to the Editor),** The Hospital, 28 & 29 Southampton Street, Strand, London, W.C.

The Cost of Subscription, post free, is as follows:—Per week, 2½d.; per quarter, 2s. 8d.; per half-year, 5s. 3d.; per annum, 10s. 6d. Foreign:—Per annum, 15s. 2d., payable, in all cases, in advance.

**NOTICE.—Vol. XXX. of "The Hospital" (April 1901, to September 1901), in handsome cloth binding can now be obtained at the office of this Journal, price, 7s. 6d. Cases for binding the Numbers contained in this Volume 1s. 6d. Index to Vol. XXX., 2½d., post free.**

**The Monthly Part for February is now ready. Price 6d., by post 8d.**

### SCRAPS AND GLEANINGS.

DURING the year 1901 over 1,200 crippled boys and girls have each spent a fortnight or month (and some a year) in the Holiday Homes of the Ragged School Union at Southend, Margate, and Bournemouth.

THE Corporation of Glasgow has given the order for the aerating machinery at Ruchill Hospital to the Volcanic Aeration Company, of Great College Street, London, the manufacturers of the "Hecla" plant.

The management of the St. Ermin's Hotel write to say that the dinner to Miss Hobhouse by the New Reform Club will not take place on their premises. The management also state that notice has been given to the New Reform Club to vacate their premises.

At the meeting of delegates of the Birmingham Hospital Saturday Fund recently held it was announced that, owing to the munificence of the Misses Stokes, the committee have an available balance of £5,000, with which they propose to buy a house with 34 acres of land for the Women's Convalescent Home, and to so alter the building as to make it capable of accommodating 80 patients. The total cost of the scheme will be about £12,000.

The Charity Festival recently held at Norwich Cathedral in aid of the Jenny Lind Infirmary was a great success, and resulted in the sum of £1,015 being placed to the credit of the endowment fund. The Chairman of the Committee of Management of the Infirmary (at a special meeting held for the purpose) proposed a vote of thanks to the Dean, and to Mrs. Lefroy, a member of their committee. This resolution was seconded by Sir Charles Gilman, and carried unanimously.

The directors of the Dumfries and Galloway Royal Infirmary, in submitting their annual report to the governors state that, in order to be in keeping with other hospitals of similar size, it has been found necessary to increase the salaries of the staff nurses. The number of in-patients during the year was 636, as against 556 last year. The treasurer's abstract of account showed a deficiency of ordinary income of only £61, the total ordinary income being £3,793 and the total ordinary expenditure being £3,854.

### The Student of Medicine.

### APPOINTMENTS.

**James Bennett,** M.R.C.S., L.R.C.P.Lond., appointed Public Vaccinator for the No. 2 District of the Warrington Union. **W. F. Bennett,** M.R.C.S., L.R.C.P., appointed District Medical Officer of the Thingoe Union. **W. J. Bannister,** M.B., appointed Junior House Surgeon to the Macclesfield Infirmary. **Arthur Cant,** M.B.. B.Ch.Birm., appointed Certifying Factory Surgeon for the Coleshill District of the County of Warwick. **D. Crowe,** M.D., B.Ch.Dub., appointed Medical Officer for the Ulceby District of the Brigg Union. **J. F. Cunningham,** L.R.C.P., M.R.C.S., appointed a House Physician at the Bethlem Royal Hospital, S.E. **Charles Fisher,** M.B., B.S.Durh., M.R.C.S., L.R.C.P.Lond., appointed House Physician to the City of London Hospital for Diseases of the Chest, Victoria Park, London. **J. Howard Fletcher,** M.R.C.S., L.R.C.P.Lond., appointed Medical Officer of Health for the Ince Urban District. **A. Leonard Fuller,** M.R.C.S Eng., L.R.C.P.Lond., appointed Anæsthetist to the Royal United Hospital, Bath. **A. K. Lauddie,** M.B.Edin., etc., appointed House Surgeon to the Wrexham Infirmary. **A. J. Little,** L.R.C.P.I., L.S.A., appointed District Medical Officer of the Uxbridge Union. **J. Allden Lloyd,** M.B.Lond., M.R.C.S., L.R.C.P.Lond., appointed House Surgeon to the Stroud General Hospital. **J. McGregor,** M.D.Glasg., appointed Medical Officer to the Kyle Union Poorhouse. **Alex. MacLennan,** M.B., C.M., appointed an Extra Honorary Surgeon to the Royal Hospital for Sick Children, Glasgow. **R. H. Meikle,** M.B., C.M.Glasg., appointed District Medical Officer of the Darlington Union. **Balfour S. Nicholson,** M.B., C.M.Glasg., appointed Medical Officer for the Parish of Clackmannan. **R. Patchett,** L.R.C.S. Edin., appointed District Medical Officer of the Clitheroe Union. **S. I. Pritchett,** M.R.C.S., L.R.C.P.Lond., appointed a Medical Officer of Health for the City and Port of Rochester, and Medical Officer to the Rochester and Chatham Joint Isolation Hospital. **James E. H. Sawyer,** M.B., M.A.Oxon., appointed a House Physician at the Bethlem Royal Hospital, S E. **R. J. H. Scott,** F.R.C.S.E., appointed Consulting Surgeon to the Royal United Hospital, Bath. **H. Stansfield,** M.B., Ch.B.Vict., appointed Medical Officer of Health for the Clayton Urban District. **D. W. M. Turpin,** L R.C.P.&S.I., L.M., appointed District Medical Officer and Public Vaccinator to the Alvaston Division of the Shardlow Union.

